# Internal fixation of acetabular quadrilateral plate fractures in elderly patients: Could the fracture reduction quality affect their functional recovery?

**DOI:** 10.1007/s40520-020-01682-1

**Published:** 2020-09-09

**Authors:** Antonello Panella, Giuseppe Solarino, Giovanni Vicenti, Davide Bizzoca, Marco Baglioni, Francesco Fortunato, Francesco Maruccia, Angela Notarnicola, Andrea Piazzolla, Raffaele Pascarella, Alberto Belluati, Biagio Moretti

**Affiliations:** 1 Orthopaedic and Trauma Unit, Department of Basic Medical Sciences, Neuroscience and Sense Organs, School of Medicine, University of Bari “Aldo Moro”, AOU Consorziale “Policlinico”, Piazza Giulio Cesare 11, 70100 Bari, Italy; 2 Department of Orthopaedic and Trauma Surgery, Ospedali Riuniti, Ancona, Italy; 3 Department of Orthopaedic and Trauma Surgery, Ravenna, Italy

**Keywords:** Quadrilateral plate, Acetabular fracture, Quality of reduction, Elderly fracture, Functional recovery, WOMAC, Harris hip score (HHS)

## Abstract

**Background:**

Osteoporotic acetabular fractures frequently involve the quadrilateral plate (QP), a flat and thin bone constituting the medial wall of the acetabulum. This study aims to assess the impact of the quality of osteoporotic QP fractures reduction on the patients’ functional recovery, at 24 months follow-up.

**Methods:**

Patients referring with osteoporotic QP fractures to our Level I trauma centre were prospectively recruited. Inclusion criteria: patients aged 60 years old or older; osteoporosis, defined as Dual-energy X-ray Absorptiometry (DXA) T-score ≤ − 2.5; acute acetabular fracture; anatomic or good fracture reduction according to Matta on postoperative CT. Exclusion criteria: moderate cognitive impairment (defined as Mini-Mental State Examination < 19); a history of malignant neoplasm; concomitant fractures in other sites; traumatic head injury; lower limb joint prostheses; patient not able to walk independently before trauma; poor fracture reduction, according to Matta, on postoperative CT. All the QP fractures were surgically managed. After surgery, the reduction of each QP fracture was classified as anatomical (displacement 0–1 mm), good (displacement 2–3 mm) and poor (displacement > 3 mm) on postoperative CT. Based on this classification: patients with a poor fracture reduction were excluded from this study, patients with an anatomical reduction were recruited in Group-A and patients with a good reduction in Group-B. All the patients underwent a clinical and radiographic 24-months follow-up.

**Results:**

68 patients (males 38; females 30; mean age 68.6 years old; range 60–79) were finally included in in the study. No cases of open fractures or concomitant pelvic ring fractures were observed. Based on the post-operative CT, 39 patients showed an anatomic fracture reduction (Group-A) while the remaining 29 patients revealed a good fracture reduction (Group-B). Complication rates and mean clinical scores showed no significant differences between groups, at 24-months follow-up.

**Conclusions:**

In this study, the functional recovery at 24 months follow-up showed no significant differences in elderly patients with QP fracture undergoing anatomical reconstruction (displacement 0–1 mm) compared to patients receiving a good QP fracture reconstruction (displacement ≤ 3 mm).

## Introduction

Acetabular fractures, with a reported annual incidence of 3 new cases over 100,000 inhabitants, are rare but challenging injuries for orthopedic surgeons [1, 2].

This kind of fractures commonly results from high-energy trauma, i.e. motor vehicle accidents, pedestrian accidents, sports injuries, and falls from a height [1]. In recent years, however, an increased incidence of acetabular fractures caused by low-energy trauma has been reported, especially in older adults with osteoporotic bone [3, 4].

Osteoporotic acetabular fractures frequently involve the quadrilateral plate (QP), a flat and thin bone constituting the medial wall of the acetabulum [5]. Isolated fractures of the quadrilateral plate are rare, thus QP injuries are generally associated with more complex fractures rimes, including both columns, anterior column, posterior hemi-transverse, posterior column, "T-shapes" or transverse fractures [6]. QP dislocation with medial femoral head migration could be also observed.

Although acetabular QP fractures represent a heterogeneous group of injuries, they mainly affect elderly patients with comparable functional requests, therefore they should be managed following the same surgical principles [7–14].

The management of acetabular fractures has radically changed in the last six decades. Until the beginning of the 1960s, most acetabular fractures were conservatively managed. In 1964, however, the principles of acetabular surgery were first described by Robert Judet and Emile Letournel, thus revolutionizing the treatment of this kind of injury [15].

Currently, the majority of authors agree the open reduction and internal fixation (ORIF) of acetabular fractures should allow early mobilization, a fast pain resolution, and an anatomic reconstruction of the hip, to prevent hip post-traumatic osteoarthritis [8, 16, 17].

It is important to note that osteoporotic acetabular fractures might be managed following different surgical principles, compared to the high-energy fractures observed in young patients. Moreover, it could be difficult to achieve the anatomical reduction and stable fixation of osteoporotic QP fractures, because of the QP location in the true pelvis and juxta-articular position, the frequent fracture comminution and the poor bone quality [18, 19].

Therefore, the definition of QP fractures reduction criteria could be useful in the surgical management of these osteoporotic fractures, to limit surgical timing and perioperative complications.

This study aims to assess the impact of the quality of osteoporotic QP fractures reduction on the patients’ functional recovery at 24 months follow-up.

## Materials and methods

### Patients selection, surgical treatment, and aftercare

Patients referring to our Level I trauma centre, between January 2010 and January 2017, with osteoporotic acetabular fractures, involving the quadrilateral plate, were prospectively recruited.

Ethical clearance was obtained from the Local Ethical Committee (Prot. n. 5556/2018), as per the 1964 Declaration of Helsinki, and all the patients gave informed consent before enrollment in the study.

Inclusion criteria: patients aged 60 years old or older; osteoporosis, defined as Dual-energy X-ray Absorptiometry (DXA) T-score ≤ -2.5; acute acetabular fracture; anatomic or good fracture reduction according to Matta on postoperative CT.

Exclusion criteria: moderate cognitive impairment (defined as Mini-Mental State Examination < 19); a history of malignant neoplasm; concomitant fractures in other sites; traumatic head injury; lower limb joint prostheses; patient not able to walk independently before trauma; poor fracture reduction, according to Matta, on postoperative CT.

Patient demographics -including age, sex, BMI-, traumatic mechanism, and fracture type according to Judet-Letournel classification [15] were recorded at recruitment. Before surgery, patients were treated with bed rest; trans-skeletal traction was used in the presence of hip instability. All the QP fractures were surgically managed within 10 days after trauma. All the patients underwent preoperative and postoperative pelvis CT scan. The DXA evaluation was performed before hospital discharge.

### Surgical procedure

All the surgical procedures were performed by the same experienced pelvic surgeon (A. Pan.) and the same anesthesiologic team. The surgical approach choice (i.e. ilioinguinal approach, modified Stoppa approach or Kocher-Langenbeck approach) and the ORIF constructs depended on the specific fracture pattern.

The ilioinguinal approach was performed with the patient in a supine position on a radiolucent table; the greater trochanter of the affected side was put at the table edge and a bump was placed under the ipsilateral buttock. The ipsilateral hip and knee were flexed, to relax the iliopsoas and the neurovascular structures.

The modified Stoppa approach was performed with the patient in a supine position on a radiolucent table. The ipsilateral limb was draped free into the surgical field and hip and knee were flexed, to relax the iliopsoas tendon and the femoral neurovascular bundle.

The Kocher-Langenbeck approach was performed with the patient in a prone position on a radiolucent table. The knee was flexed at 90° and the hip extended, to reduce the intraoperative tension on the sciatic nerve.

After surgery, the patients observed bed rest for twenty days, then partial weight-bearing was prescribed for the following 8 weeks.

### Post-operative CT evaluation

Two orthopedic surgeons, with more than 5 years of experience in hip and pelvis surgery and not involved in the surgical procedure, evaluated the quality of QP fracture reduction and the joint congruence on post-operative CT, using Matta’s criteria [20].

The reduction of each QP fracture was classified as anatomical (displacement 0–1 mm), good (displacement 2–3 mm), and poor (displacement > 3 mm). Based on this classification, patients with a poor fracture reduction were excluded from this study, patients with an anatomical reduction were recruited in Group-A and patients with a good reduction in Group-B. All the patients underwent a clinical and radiographic 24-months follow-up.

### Clinical and radiological assessment at follow-up

Patients underwent a clinical and radiological follow-up at two-, six-, twelve-, eighteen, and twenty-four months post-operatively. Complications and reoperations were recorded.

Clinical evaluation was performed using the following validated scores: Harris Hip Score (HHS), modified Merle D’Aubigné-Postel Score (MMDAPS) [21] and the Western Ontario and McMaster Universities Osteoarthritis Index (WOMAC) [22].

At each follow-up, all the patients underwent a radiographic evaluation of the pelvis, including anteroposterior view and Judet views (iliac oblique and obturator views). On each X-ray, the following elements were evaluated: the quality of fracture reduction; the fracture healing process; the presence of heterotopic calcifications (classified according to Broker [24]) and the presence of femoral head avascular necrosis (classified according to Ficat and Arlet [26]).

The pelvis X-rays performed at the 24 months follow-up were also checked for hip osteoarthritis according to Matta’s criteria [20].

### Statistical analysis

Statistical analysis was performed by two authors (A.N., M.B.,) using STATA/MP 14 for Windows (StataCorp LP, College Station, USA). All the data were described as mean, median, and standard deviation.

Categorical variables were evaluated as absolute frequencies and proportions. The proportions in the two groups were compared using the chi-square test. Continuous variables were described as means; differences between the two groups were evaluated using the Wilcoxon-Mann–Whitney test for independent samples. A *p*-value < 0.05 was considered statistically significant.

## Results

The main data of the study are summarized in Table 1. 72 patients were originally included in the study, but 4 patients out of 72 were lost to follow-up (drop-out 5.56%). Therefore, 68 patients (males 38; females 30; mean age 68.6 years old; range 60–79) were finally included in the study. No cases of open fractures or concomitant pelvic ring fractures were found.

**Table 1:** Main data of the study

	Group-A (Anatomical reduction)	Group-B (Good reduction)
No of patients	39	29
Age		
Mean ± SD	67.52 ± 5.88	64.43 ± 8.24
Range	60–79	62–77
Gender		
Male, *n* (%)	21 (53.85%)	18 (62.07%)
Female, *n* (%)	18 (46.15%)	11 (37.93%)
BMI (Kg/m^2^)		
Mean ± SD	28.3 ± 1.76	27.6 ± 1.48
Side		
Left, *n* (%)	25 (64.1%)	17 (58.62%)
Right, *n* (%)	14 (35.9%)	12 (41.83%)
Mechanism of injury		
Motor vehicle accident, *n* (%)	9 (23.08%)	7 (24.14%)
Pedestrian accident, n (%)	3 (7.39%)	2 (6.9%)
Fall from a height, n (%)	8 (20.5%)	5 (17.24%)
Simple fall, n (%)	19 (48.72%)	15 (51.72%)
Judet-Letorunel classification		
Quadrilateral plate alone, *n* (%)	1 (2.56%)	–
Anterior column, *n* (%)	3 (7.69%)	4 (13.8%)
Both columns, *n* (%)	5 (12.8%)	3 (10.34%)
Hemi-transverse, *n* (%)	4 (10.26%)	3 (10.34%)
Transverse, *n* (%)	75 (12.8%)	3 (10.34%)
Transverse + Anterior wall, *n* (%)	8 (20.5%)	5 (17.24%)
Hemitransverse + Anterior column, *n* (%)	7 (17.95%)	5 (17.24%)
Transverse + Anterior column, *n* (%)	6 (15.44%)	6 (20.7%)
Surgical approach		
Ilioinguinal	10 (25.64%)	10 (34.48%)
Ilioinguinal + modified Stoppa	24 (61.54%)	16 (55.18%)
Ilioinguinal + Kocher-Langenbeck	5 (12.8%)	3 (10.34%)
DXA T-score		
Mean ± SD	−3.6 ± 0.8	−3.75 ± 0.74

Based on the post-operative CT scans, 39 patients showed an anatomic fracture reduction (Group-A) while the remaining 29 patients revealed a good fracture reduction (Group-B).

Depending on the specific fracture patterns (Table 1), different ORIF constructs were used: in 22 patients out of 68 (32.36%) a reconstruction plate of the anterior column alone was used; in 30 patients out of 68 (44.12%) a QP plating was performed in addition to the anterior column plating; in 8 patients out of 68 (11.76%), affected by both columns fracture, an ORIF of either anterior and posterior columns was performed; in 8 patients of 68 (11.76%), an infrapectineal plate was used in addition to an anterior column reconstruction plate.

The complications observed during the 24 months follow-up are summarized in Table 2. No significant differences between groups were observed (Table 2).

**Table 2:** Late complications observed during the 24 months follow-up: comparison between groups (Chi-square test; statistical significance was set at *p* < 0.05)

	Group-A (Anatomical reduction)	Group-B (Good reduction)	*p*
Early complications			
Lateral femoral cutaneous nerve injury, *n* (%)	2 (5.13%)	1 (3.45%)	0.065
Intra-operative vascular injuries, *n* (%)	1 (2.56%)	1 (3.45%)	0.093
Pulmonary embolism, *n* (%)	1 (2.56%)	1 (3.45%)	0.093
Surgical Site Infections, *n* (%)	–	–	–
Late complications			
*Heterotopic ossification*			
Broker I, *n* (%)	16 (41.03%)	12 (41.38%)	0.643
Broker II, *n* (%)	7 (17.95%)	5 (17.24%)	0.532
Broker III, *n* (%)	3 (7.69%)	2 (6.9%)	0.087
Avascular femoral head necrosis			
Ficat 0, *n* (%)	7 (17.95%)	5 (17.24%)	0.532
Ficat I, *n* (%)	3 (7.69%)	2 (6.9%)	0.087
Ficat IIA, *n* (%)	4 (10.26%)	3 (10.34%)	0.84
Ficat IIB, *n* (%)	3 (7.69%)	2 (6.9%)	0.087
Hip Osteoarthritis (Matta)			
Excellent, *n* (%)	8 (20.5%)	6 (20.68%)	0.865
Good, *n* (%)	17 (43.6%)	12 (41.37%)	0.0922
Sufficient, *n* (%)	13 (33.34%)	10 (34.5%)	0.211
Poor, *n* (%)	1 (2.56%)	1 (3.45%)	0.093

*p < 0.05

Table 3 shows the clinical scores recorded in both groups at 24 months follow-up. No significant differences were depicted (Table 3).

**Table 3:** Clinical scores at 24 months follow-up: comparison between groups (Wilcoxon-Mann–Whitney test for independent samples)

	Group-A	Group-B	*p*
HHS	85.7 ± 9.3	85.8 ± 9.6	0.96
MMDAPS	15.6 ± 1.6	15.8 ± 1.3	0.69
WOMAC	14.2 ± 6.1	12.3 ± 5.1	0.34

Statistical significance was set at *p* < 0.05

## Discussion

In recent years, an increased prevalence of acetabular fractures has been observed in the elderly, because of the general population aging [21].

Osteoporotic acetabular fractures commonly involve the QP and should be managed following different surgical principles, compared to traumatic acetabular fractures [3]. Hence, low-energy trauma, poor bone quality, the subsequent limited opportunities for screw purchase, relatively low functional demand and recovery expectation are the main features differentiating osteoporotic acetabular fractures from the traumatic ones [3].

Anatomical reduction and stable fixation play a key role in the management of articular fractures, to restore the joint congruity, thus reducing the risk of post-traumatic osteoarthritis [22]. In the surgical management of acetabular fractures, Letournel and Matta have shown the anatomical reduction of the fracture is one of the leading factors influencing the final clinical outcome [23]. Hence, a poor fracture reduction, i.e. a displacement greater than 3 mm, is currently considered a negative prognostic factor for the final functional outcome [23].

Nonetheless, none of the previous studies, to the best of the authors’ knowledge, has focused on the definition of osteoporotic QP fractures reduction criteria.

In this prospective study, 68 patients with osteoporotic QP fractures were included to assess if the quality of QP fracture reduction could influence the patients’ functional recovery at 24 months follow-up. The recruited patients, based on the postoperative pelvis CT, were divided into two groups: 39 patients with an anatomical reduction of the QP fracture (displacement 0–1 mm) were recruited in Group-A, whereas the remaining 29 patients, showing a good fracture reduction (displacement 2–3 mm) were recruited in Group-B.

We used postoperative pelvis CT to assess the fracture reduction quality since, as suggested by Matta et al. in 1996 [24] and subsequently confirmed by Moed et al. [25], plain radiographs are not enough accurate in demonstrating articular incongruities.

The surgical approach choice (i.e. ilioinguinal approach, modified Stoppa approach or Kocher-Langenbeck approach) depended on the specific fracture pattern, according to the literature [7, 26–28]. The ilioinguinal approach alone was performed in 20 patients out of 68 (29.41%), ilioinguinal approach and concomitant Kocher-Langenbeck approach were performed in 8 patients out of 68 (11.76%) while ilioinguinal approach and concomitant modified Stoppa approach were performed in 40 patients out of 68 (58.82%).

The use of a modified Stoppa approach in the ORIF of QP fractures was suggested by Cole et al. [26], to obtain a better intrapelvic view. These authors evaluated 55 patients undergoing ORIF for QP fractures and, at a mean follow-up of 17.7 months, an excellent modified Merle D’Aubigné-Postel score was recorded in 47% of cases and an excellent Matta radiographic score was observed in 64% of cases. More recently, similar results have been reported by Yang et al. [27] and Laflamme et al. [7] in patients with QP fractures undergoing open reduction and internal fixation.

Interestingly, in the present study, early and late complications rates, as well as the mean clinical scores recorded at 24 months follow-up showed no significant differences between groups. Hence, a postoperative fracture displacement ≤ 3 mm, assessed on CT, could guarantee a good clinical outcome at 24 months follow-up, in elderly patients with QP fractures.

Acute total hip arthroplasty (THA) has been also proposed in the management of osteoporotic acetabular fractures [28–30]. Although aseptic acetabular loosening and THA dislocation have been reported in patients undergoing acute THA for acetabular fractures, Trabecular Metal (TM) revision acetabular shells have recently shown promising results, since they have revealed effective in enhancing bone ingrowth and fixation and have provided good clinical outcomes [30].

This study, on the other hand, supports the ORIF of osteoporotic QP fractures. Moreover, in the present study, a postoperative fracture displacement ≤ 3 mm provided the same clinical results as the anatomical reduction. This is a relevant finding since QP fractures are high-demanding injuries and the anatomical reduction of these fractures is often difficult to be reached. Consequently, this study might positively influence our daily clinical practice, thus, reducing surgical timing and, hopefully, the incidence of intraoperative and postoperative complications.

The main strengths of our study are the relatively long follow-up and the conspicuous patients’ sample, consistent with the frequency of osteoporotic QP fractures.

However, the limits of the present study could not be overcome, including the heterogeneity of the surgical approaches performed and the different plating constructs employed. Furthermore, the lack of pelvis CT scans performed during the different follow-ups is another limitation of the present study.

## Conclusions

**Fig. 1:**
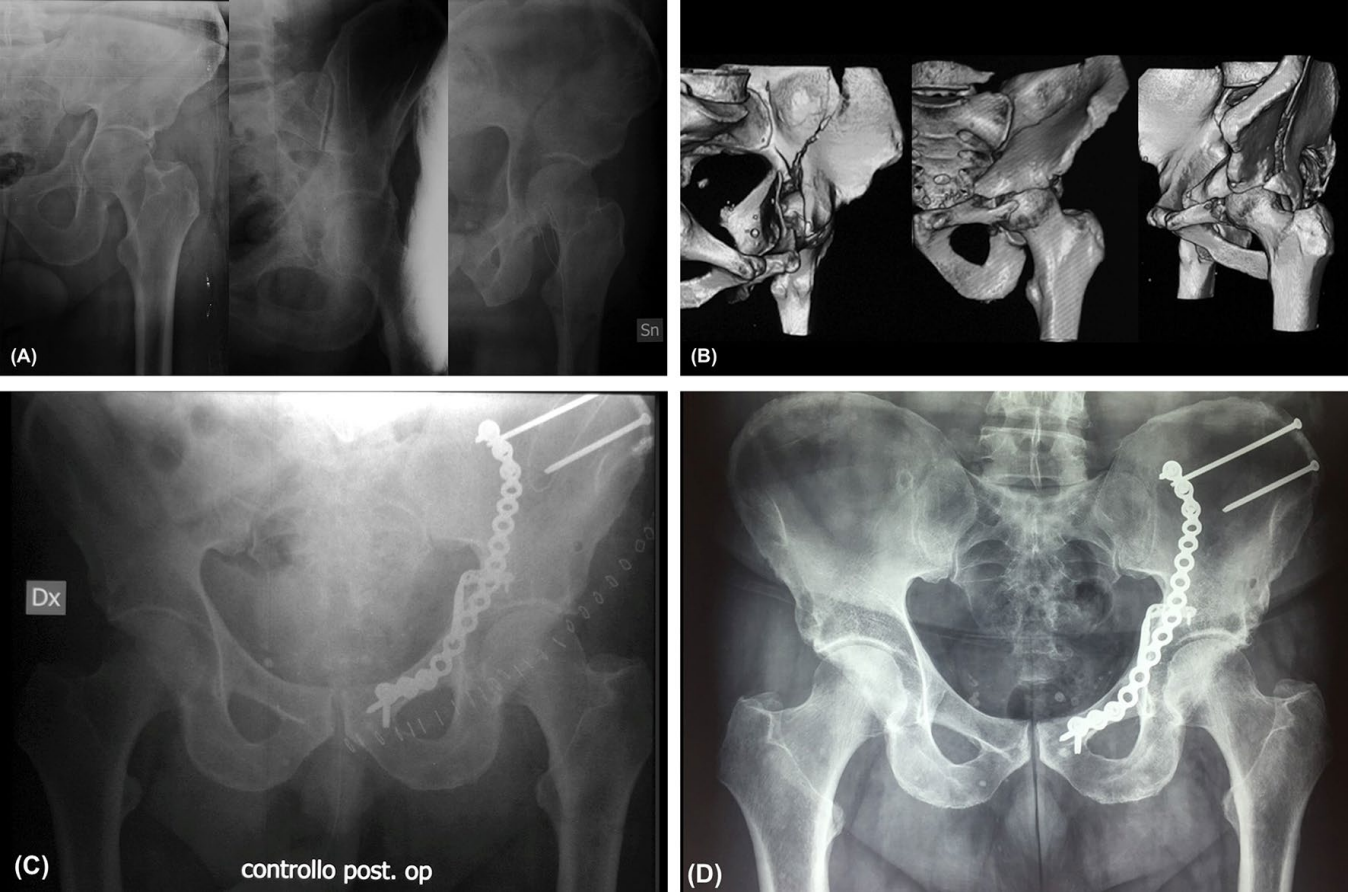
A 61 years old man reported, in a car accident, a left hemitransverse + anterior column acetabular fracture. **a** Preoperative X-rays: AP view and Judet views. **b** Preoperative CT.** c** Postoperative X-rays. **d** X rays at 24 months follow-up

In this prospective case series, the functional recovery at 24-months follow-up showed no significant differences in patients with osteoporotic QP fractures receiving an anatomical reduction (displacement 0–1 mm) compared with patients receiving good a fracture reduction (displacement ≤ 3 mm). Therefore, the anatomical reduction is not strictly needed in the open reduction and internal fixation of osteoporotic QP fractures (Fig. 1).
